# Dual Resonant Frequencies Effects on an Induction-Based Oil Palm Fruit Sensor

**DOI:** 10.3390/s141121923

**Published:** 2014-11-19

**Authors:** Noor Hasmiza Harun, Norhisam Misron, Roslina Mohd Sidek, Ishak Aris, Hiroyuki Wakiwaka, Kunihisa Tashiro

**Affiliations:** 1 Faculty of Engineering, University Putra Malaysia, Serdang 43400, Selangor, Malaysia; E-Mails: noorhasmiza@unikl.edu.my (N.H.H.); roslinams@upm.edu.my (R.M.S.); ishak_ar@upm.edu.my (I.A.); 2 Institute of Advanced Technology (ITMA), University Putra Malaysia, Serdang 43400, Selangor, Malaysia; 3 University Kuala Lumpur-British Malaysian Institute, Batu 8, Jalan Sg Pusu, Gombak 53100, Selangor, Malaysia; 4 Faculty of Engineering, Shinshu University, Wakasato 4-17-1, Nagano 380-8553, Japan; E-Mails: wakiwak@shinshu-u.ac.jp (H.W.); tashiro@shinshu-u.ac.jp (K.T.)

**Keywords:** induction, resonant frequency, air coil, oil palm, frequency characteristics, maturity classification, inductive concepts

## Abstract

As the main exporter in the oil palm industry, the need to improve the quality of palm oil has become the main interest among all the palm oil millers in Malaysia. To produce good quality palm oil, it is important for the miller to harvest a good oil palm Fresh Fruit Bunch (FFB). Conventionally, the main reference used by Malaysian harvesters is the manual grading standard published by the Malaysian Palm Oil Board (MPOB). A good oil palm FFB consists of all matured fruitlets, aged between 18 to 21 weeks of antheses (WAA). To expedite the harvesting process, it is crucial to implement an automated detection system for determining the maturity of the oil palm FFB. Various automated detection methods have been proposed by researchers in the field to replace the conventional method. In our preliminary study, a novel oil palm fruit sensor to detect the maturity of oil palm fruit bunch was proposed. The design of the proposed air coil sensor based on the inductive sensor was further investigated mainly in the context of the effect of coil diameter to improve its sensitivity. In this paper, the sensitivity of the inductive sensor was further examined with a dual flat-type shape of air coil. The dual air coils were tested on fifteen samples of fruitlet from two categories, namely ripe and unripe. Samples were tested within 20 Hz to 10 MHz while evaluations on both peaks were done separately before the gap between peaks was analyzed. A comparative analysis was conducted to investigate the improvement in sensitivity of the induction-based oil palm fruit sensor as compared to previous works. Results from the comparative study proved that the inductive sensor using a dual flat-type shape air coil has improved by up to 167%. This provides an indication in the improvement in the coil sensitivity of the palm oil fruit sensor based on the induction concept.

## 1. Introduction

The oil palm constitutes the largest oil crop in Malaysia, with an average annual production of 4.0 metric tonnes of palm oil per hectare of land. To ensure that maximum amount of palm oil is extracted from the mesocarp, it is important that the oil palm FFBs are harvested at the correct stage of ripeness [1,2]. In Malaysia, the human expert grading approach is used to inspect the maturity of the oil palm FFBs and classify them for harvesting. The color of the surface of the fruit and the number of loose fruit dropping from bunches are left to the judgment of human experts [2,3]. In practice, this grading method has a high potential for grading the fruit incorrectly. Furthermore, the potential of obtaining different grading results between the human graders is high. Apart from that, visual inspection by the human grader is a time-consuming method and often leads to considerable profit losses [4,5]. Therefore, an automated fruit grading system is highly demanded. An ideal automated fruit grading system should be rapid, accurate and reliable [6–8].

In the past few years, various automated fruit grading systems were proposed and tested by Malaysian researchers. The most common method is the color vision system, which uses an advanced digital camera to collect a picture of oil palm FFBs and a computer setup for further analysis [9–12]. Added to this, an artificial intelligence system is commonly used with the color vision system to classify the oil palm FFBs [13–20]. Another common color vision system is the assessment using RGB space. This assessment method uses spectral analysis based on different wavelengths of the red, green and blue colors of the captured image [21,22]. Relatively, the color quality of the image is important in this method. However, the main drawback of this method is that it has to be performed indoors [23–25].

The moisture content of oil palm is another grading assessment that is quite popular among researchers. The moisture content of the mesocarp in the fruit plays a major role that affects the surface color and the weight of the oil palm fruit. In this method, a microwave moisture sensor is used to measure the moisture content of oil palm. This procedure is quite complicated and time-consuming [26–30].

As for the imaging method, the most popular techniques used are Magnetic Resonance Imaging (MRI) and bulk Nuclear Magnetic Resonance (NMR) [31]. The monitoring system looks into the differences in their spin-spin relaxation times between oil and moisture content in the oil palm FFBs. Non-destructive Infra-red (NIR) spectroscopy is another equipment used in imaging assessment. The assessment consists of a pair of NIR spectrometers, which is used to scan the oil palm fruits in different modes. Then, the analysis of the chemical contents of palm oil is assisted with the Partial Least Squares Regression (PLSR) models [32]. Apart from complicated and expensive equipment, skilled personnel are needed to operate it, and the imaging assessment process is confined indoors.

Another grading assessment technique currently considered is a capacitive-based assessment. The capacitive-based grading method was proposed in [33]. It measures the dielectric properties of the oil palm fruit. A comparative analysis on the dielectric properties, ε, will summarize the analysis. Again, the supporting equipment needed for the assessment limits the outdoor testing possibilities.

A novel grading method based on the induction concept is proposed as it is not on the prevailing research list yet. The induction-based oil palm fruit sensor highlights the resonant frequency of its air coil. The primary design of the air coil, the ring-type shape air coil affects its frequency characteristics, specifically the effects of coil's diameter were studied in the preliminary research work [34–36]. The research continues with a new air coil structure design, where flat-type shaped air coils with various air coil lengths and coil diameters were studied. The main concern was to improve the sensitivity of the inductive oil palm sensor in determining the maturity of the oil palm FFB. The result showed that the 5 mm air coil length with 0.12 mm coil diameter has the highest sensitivity value [35]. The promising results from both research works helped improve the potential of the proposed induction-based sensor to be used for outdoor testing [34,35].

In this paper, further investigation on the flat-type shape air coil of the induction-based oil palm fruit sensor is presented. Two flat-shape air coils with different numbers of turns were used in the testing. Three different combinations; 180-140 turns, 200-140 turns and 250-140 turns were tested on fifteen palm fruit samples from two categories: ripe and unripe fruitlet. The result evaluation methods were the same as used in [34,35]. As in theory, each air coil initiated its own resonant peak. Thus, the resonant frequency (*f*_r_) characteristics of each coil's turns configuration provided two resonant peaks. The first and the highest peak came from the air coil with the highest number of turns of each combination. As for the second peak, the inductance value was lower than that of the first peak and its *f*_r_ approached 10 MHz. The analysis started with the value of the *f*_r_ of air and both samples from both peaks being normalized to the resonant frequency of air (*f*_ra_). Then, the average value of each normalized resonant frequency (N*f*_r_) was calculated. For further analysis, the difference between the N*f*_r_ of both samples to the normalized frequency of air (N*f*_ra_) was calculated. The ratio between δ_unripe_ (the difference between N*f*_ra_ and N*f*_ru_) and δ_ripe_ (the difference between N*f*_ra_ and N*f*_rr_) was analyzed at the end of each peak's analysis. In this paper, the improvement in sensitivity of the sensor was determined by a comparative analysis done at the end of the peak evaluation based on the previous work [35]. At the beginning, peak analysis was conducted separately where the first peak shows that the 200-140 coil turns version has the highest ratio value, whereas in the second peak analysis, the 250-140 coil turns system turned out to be the best. Meanwhile, the gap between both peaks evaluated the 200-140 coil turns showed the highest value of the ratio between δ_unripe_ and δ_ripe_. Finally, results from the comparative analysis proved that the induction-based oil palm fruit sensor using a dual flat-type shape air coil has improved its sensitivity up to 371% in terms of differences between the normalized sample mean from the previous works [35]. As for the ratio between δ_unripe_ and δ_ripe_, the dual flat type shape air coil showed an improvement by 236% as compared to [35]. The 200-140 coil turns configuration turned out to be the best among the three configurations and the first peak is believed to be the most stable and promising peak to be used in the analysis.

## 2. Basic Principles of Dual Resonant Frequencies

### 2.1. Structure

**Figure 1.**
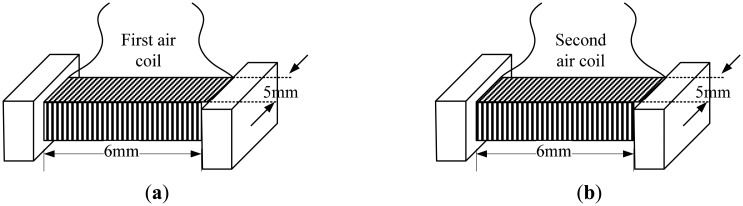
Flat-type shape air coil (**a**) First air coil, (**b**) Second air coil.

The general structure of the flat-type shape air coil is shown in Figure 1. The flat 5 mm × 6 mm surface is the main platform where copper wire with a diameter of 0.12 mm is wound around it. Three configurations in terms of number of turns were used, namely 180-140 turns, 200-140 turns and 250-140 turns. To hold the air coil tight and without displacement, it was placed in a holder. All parts in the inductive based oil palm fruit sensor involving the air coil and its holder are made of the ABS filament. The ABS filament (used as cartridge for the 3D printer) is a non-conducting material that minimizes the flux disturbance in the sensor. Table 1 shows the coil turn configurations for the dual flat type shape air coil. To minimize the errors when measurements are taken, the winding of the copper coil was kept as close as possible, reducing the gaps between winding.

### 2.2. Measurement Setup

**Figure 2.**
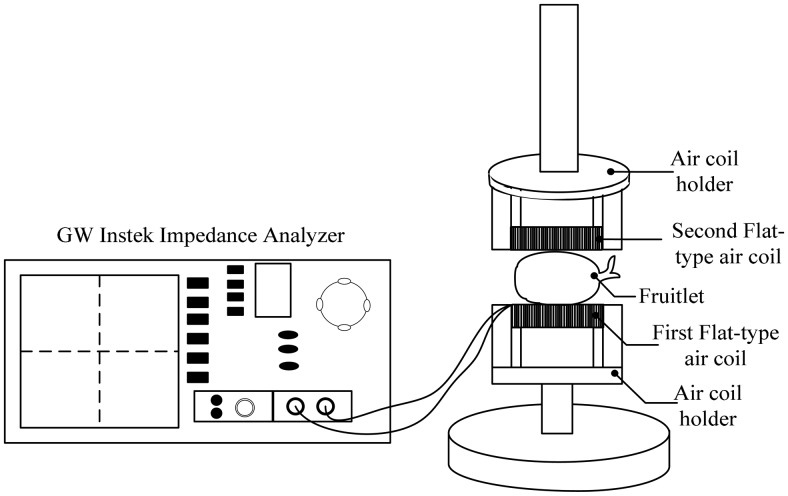
Experimental setup for the flat-type air coil.

**Figure 3.**
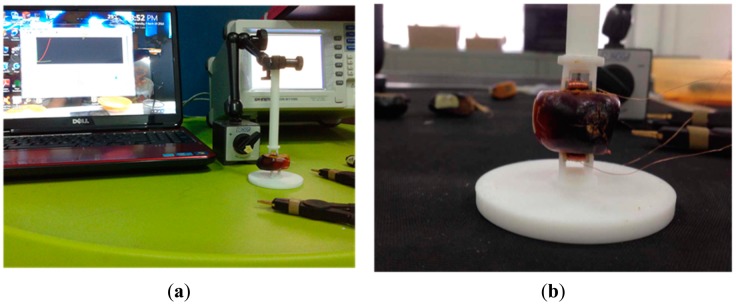
Samples preparation for the dual flat-type shape air coil. (**a**) Experimental setup, (**b**) Sample close up.

The investigation started with the measurement arrangement depicted in Figure 2. Thirty samples of fruitlets from two categories—ripe and unripe—were tested between 20 Hz to 10 MHz. A GW Instek (LCR 8110G) impedance analyzer (Good Will Instrument Co., Taipei, Taiwan) was used to measure the inductance, the resistance and the resonant frequency of the air coil for each configurations. Figure 3a shows the experimental setup used in the dual flat type shape air coil, where the gap between the fruit and the sensor was minimized in order to get precise results. To this end the sample was cut on both sides facing the air coil as in Figure 3b so that the surface contact with the air coil is flat and no gap exists between the fruit and the sensor.

**Figure 4.**
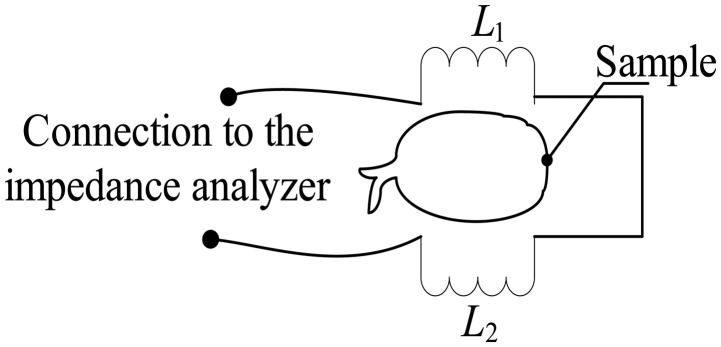
Electrical diagram for the dual flat type shape air coil.

Figure 4 shows the electrical diagram of the dual flat type shape air coil, which is connected in series. *L*_1_ represents the first air coil's inductance and *L*_2_ as the second air coil's inductance.

**Figure 5.**
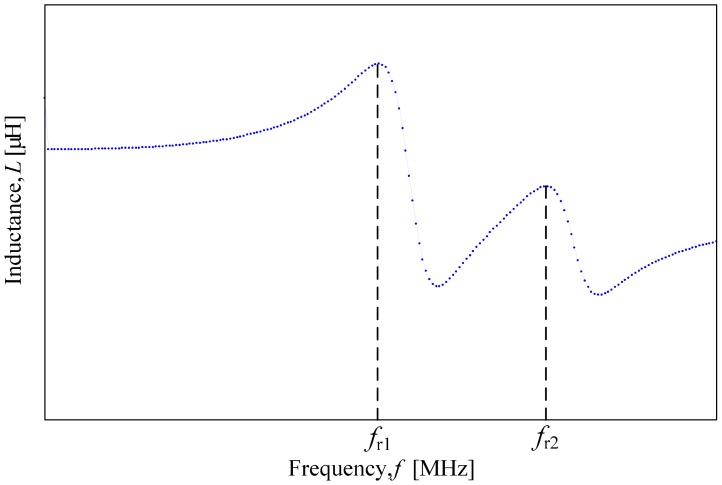
Expected dual resonant frequencies.

The GW Instek (LCR 8110 G) impedance analyzer with the setup as in Table 2 was standardized throughout the measurement period as well as for all sample and coil turn configurations. Figure 5 shows the expected dual resonant frequencies graph, which was obtained from the measurement. There are two peaks in the graph; the first peak results from the air coil with the highest number of turns and the second peak is *vice versa*.

## 3. Methodology

### 3.1. Selection of Samples

Selection of samples and category was basically similar to previous works [34,35]. Thirty samples from two categories were selected, namely ripe and unripe. Standard specifications used by the Malaysian Palm Oil Board were used in selecting samples such as the surface color of the fruitlet and its age. The selected unripe sample was dark purple in color, whereas the ripe sample was orange in color. For the unripe fruitlet, the samples were selected after the 7th week after anthesis (WAA) and for the ripe fruitlet after the 18th week after anthesis (WAA) [34]. Each sample was selected and freshly plucked from the same oil palm Fresh Fruit Bunch (FFB) on the day of testing. Table 3 summarizes the characteristics of the selected samples in this study.

### 3.2. Evaluation Method

**Figure 6.**
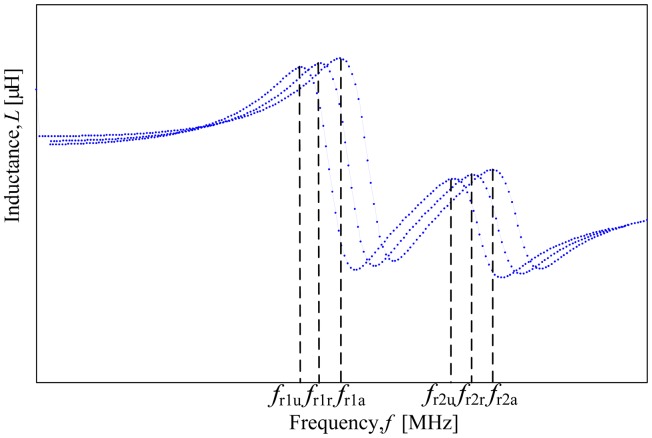
Peaks labeling for all resonant frequencies.

The evaluation methods used in this study were adopted from previous works. Initially, all resonant frequencies are normalized to their *f*_ra_. Figure 6 explains in detail the normalization process of the resonant frequency. Detailed calculations of the normalized frequency for air, ripe fruit and unripe fruit of each peaks are shown in Equations (1) through (8) in Table 4. Further evaluations were conducted using the normalized resonant frequencies specifically in the ratio between the difference in the mean sample for N*f*_ra_, with the difference in the mean sample for N*f*_rr_ and N*f*_ru_ from both peaks.

Figure 6 illustrates dual resonant frequency labeling used in this paper. The resonant frequency of air of the first peak is designated as *f_r_*_1_*_a_* whilst *f_r_*_2_*_a_* is the resonant frequency of air of the second peak. As for the ripe samples, *f_r_*_1_*_r_* is labeled as the resonant frequency of ripe sample of the first peak and *f_r_*_2_*_r_* as the resonant frequency of ripe sample of the second peak. Similarly, *f_r_*_1_*_u_* is used as the symbol for the resonant frequency of unripe sample of the first peak and *f_r_*_2_*_u_* is the resonant frequency of unripe sample of the second peak.

## 4. The Dual Resonant Characteristics

**Figure 7.**
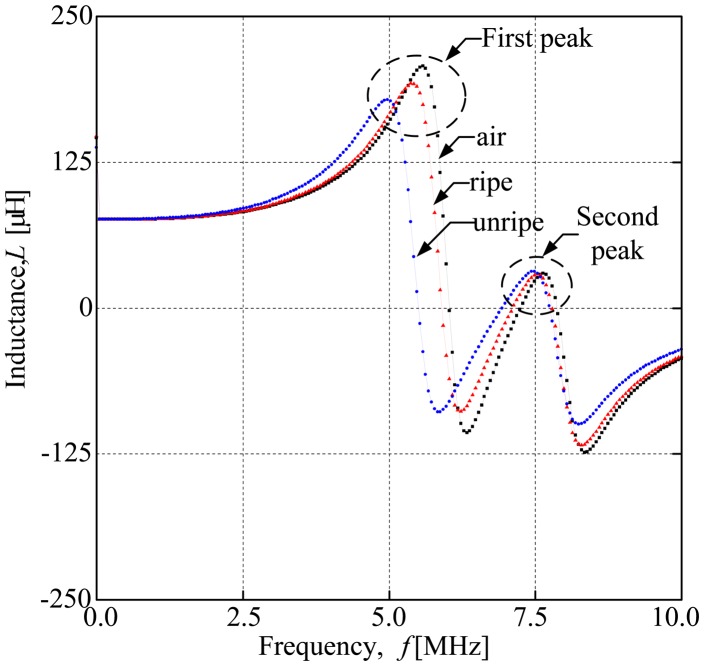
Inductance characteristics of the oil palm fruit sensor.

When analyzing the inductance characteristic of the air, ripe fruit and unripe fruit, they give different resonant frequencies, where the resonant frequency refers to the peak of the frequency. Although air, ripe fruit and unripe fruit give different resonant frequencies, they are on the same curves where the resonant frequency of air, *f*_ra_ leads the resonant frequency of ripe sample, *f*_rr_ and *f*_ru_ (the resonant frequency of unripe sample). Figure 7 shows the general inductance characteristic of the air coil when it is running within a range of frequency 20 MHz–10 MHz. In this testing, there were two resonant peaks stemming from both air coils having different number of turns. The first and the highest peak came from the air coil with a lower number of turns, whilst, the second and the lowest peak came from the second air coil with the highest number of turns from each configuration. In Figure 7, the first peak seems to dominate the testing results. The first peak offered a distinctive resonant peak between samples and established the result for the second peak. Therefore, the first peak plays an important role in further analysis in this paper. This dual inductance characteristics of the air coil portrays a similar pattern throughout the whole series of experiments. To secure the repeatability of the sensor, the dual inductance characteristics of each air coil should be standardized. The basic evaluation method used in this paper is the normalization of each resonant frequency to the resonant frequency of air, *f*_ra_. Then, the average value of the normalized resonant frequency was calculated before the difference between the value is counted. Finally, the ratio between the differences was obtained and analyzed. The ratio was further analyzed between peaks to evaluate its improvement in terms of sensitivity as compared to the previous work.

### 4.1. Characteristics of the First Peak, f_r1_

**Figure 8.**
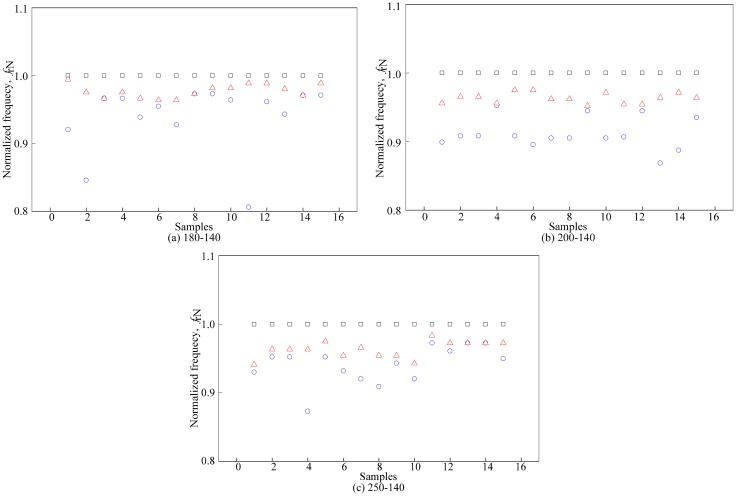
Normalized resonant frequency of the first peak for each coil's turns configuration. □ air 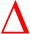
ripe 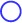 unripe.

The experiments conducted for all configurations of the dual flat-type shape air coil were aimed at collecting the resonant characteristics of the sensor. The resonant frequency was then plotted against the samples. The values of the resonant frequency of fruitlet samples, *f*_r_ from both air coils were then normalized to the value of the resonant frequency of air, *f*_r1a_. The graph in Figure 8 shows a similar pattern for all samples, regardless of the turns configurations. Generally, the resonant frequency of air, *f*_rair_ leads the resonant frequency of ripe, *f*_rr_ and unripe samples, *f*_ru_. To clarify the observation, the resonant frequency for each turn's configuration was then normalized to the resonant frequency of air from the first peak, *f*_r1a_.

**Figure 9.**
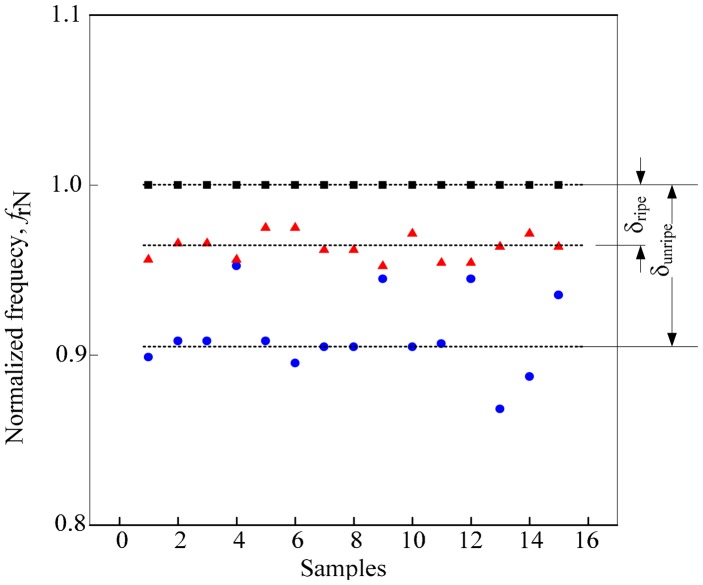
Calculated average value of the normalized resonant frequency.

**Figure 10.**
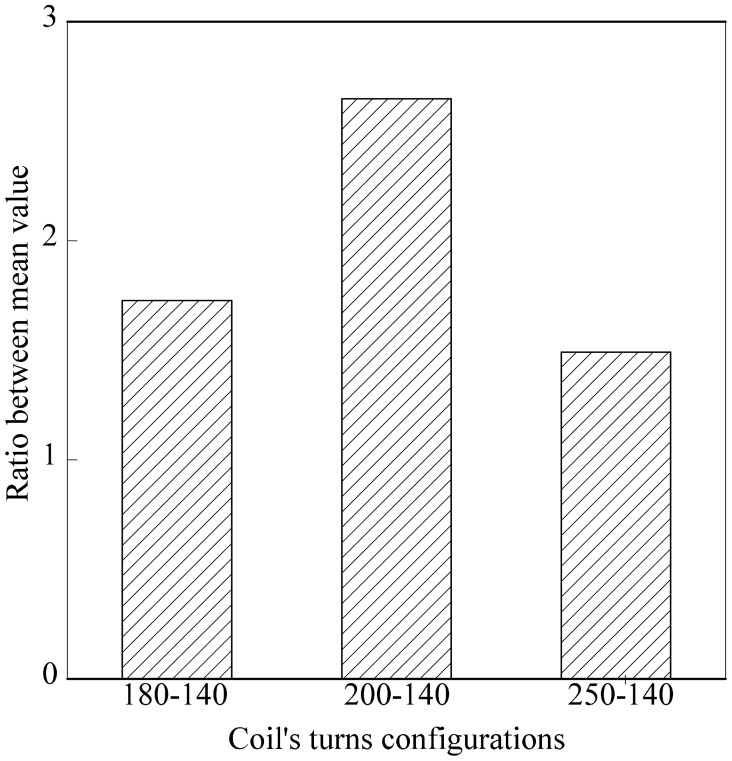
Ratio between δ_unripe_ and δ_ripe_ for each coil turn configuration.

The evaluations started when the average value of each normalized resonant frequency is calculated. The dotted line in Figure 9 represents the sample mean of the N*f*_rair_, N*f*_rr_ and N*f*_ru_. Then, the difference between the $\bar{Nf_{r1a}}$ and $\bar{Nf_{r1r}}$designated as δ_ripe_ and δ_unripe_ as the difference between $\bar{Nf_{r1a}}$ and $\bar{Nf_{r1u}}$ were obtained. An ANOVA analysis using SPSS was conducted to calculate the value of the standard deviation and variance for ripe and unripe fruits *vs.* air. The value from the ANOVA analysis is presented in Table 5. On the other hand, the ratio between δ_unripe_ and δ_ripe_ was calculated for each coil's turns configuration and presented in Table 6. Detailed values in Table 6 are presented visually in Figure 10. Results from the graph indicated that the air coil with 200-140 coil turns provided the highest ratio between δ_unripe_ and δ_ripe_.

### 4.2. Characteristics of the Second Peak, f_r2_

**Figure 11.**
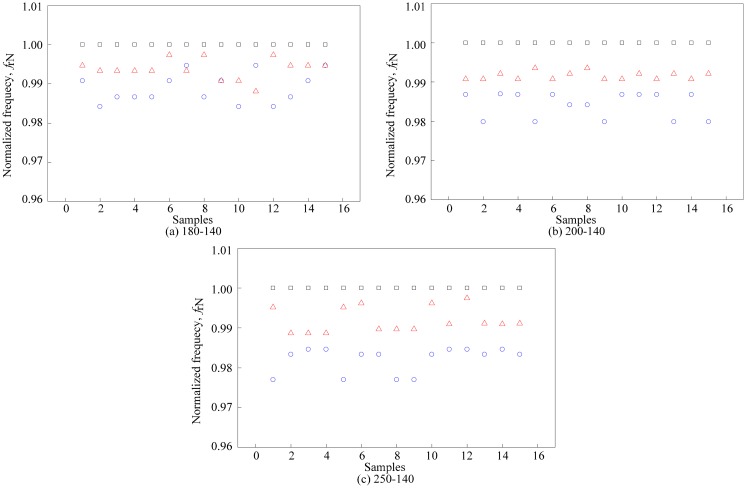
Normalized resonant frequency of the second peak for each coil's turns configuration: □ air 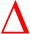 ripe 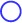 unripe.

Figure 11 shows the normalized resonant frequency of the second peak of each coil turn configuration. The results show similar patterns as the first peak except that the readings between samples are slightly unstable. This is attributed to the dual resonant frequencies effects where the first peak dominates the overall performance of the sensor.

**Figure 12.**
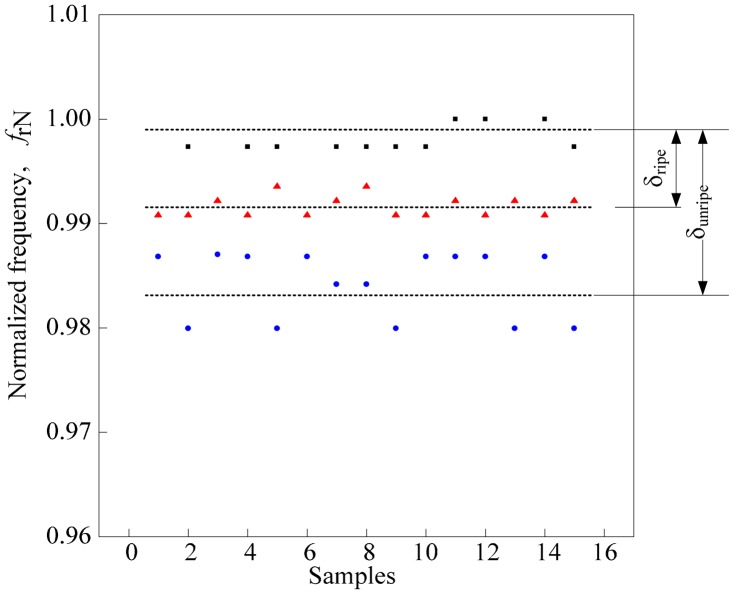
Calculated average value of the normalized resonant frequency.

**Figure 13.**
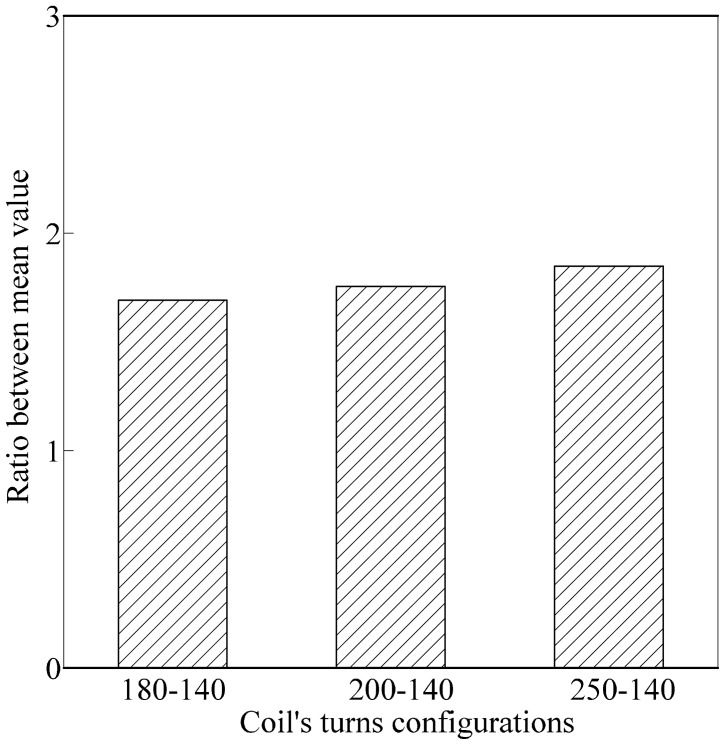
Ratio between δ_unripe_ and δ_ripe_ for each coil turn configuration.

Similarly, in Section 4.1, the normalized resonant frequency of air, N*f*_r2a_ leads the normalized resonant frequency for ripe N*f*_r2r_, and unripe N*f*_r2u_ samples. Despite of the minor flaws seen in the normalized resonant graph in Figure 11, a similar evaluation method as in Section 4.1 is used on the second peak's results. Basically, the resonant frequency at the second peak, *f*_r2_ is normalized to *f*_r2a_. Then, the sample mean of each sample is calculated along with the difference between samples and air as shown in Figure 12. The sample mean of the N*f*_r2a_, $\bar{Nf_{r2a}}$ and both samples ( $\bar{Nf_{r2r}}$ and $\bar{Nf_{r2u}}$ ) are represented as a dotted line. Then, the difference between the $\bar{Nf_{r2a}}$ and both samples ( $\bar{Nf_{r2r}}$ and $\bar{Nf_{r2u}}$ ) are computed and designated as δ_unripe_ and δ_ripe_, respectively. Line in Section 4.1, an ANOVA analysis using SPSS was conducted to calculate the value of standard deviation and variance for ripe and unripe fruits *vs.* air. The value from the ANOVA analysis is presented in Table 7. As for the average value results, the ratio between δ_unripe_ and δ_ripe_ is calculated and presented in Table 8. The results in Table 8 are illustrated in Figure 13. From the graph, the ratio between δ_unripe_ and δ_ripe_ for each coil turn configuration increases in small increments. In this analysis, the air coil with 250-140 coil turn configuration provides the highest value of the ratio. However, as discussed in Section 4.1, readings obtained from the second peak are slightly unstable, resulting in dissimilar patterns as noted from the first peak's analysis.

### 4.3. Dual Peaks Analysis

**Figure 14.**
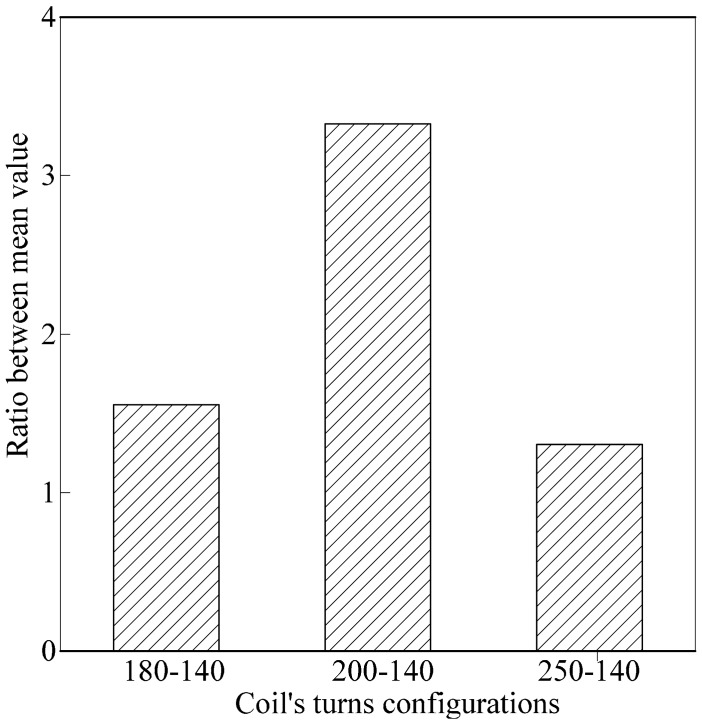
Ratio between δ_unripe_ and δ_ripe_ for dual peaks.

Further analysis on the ratio evaluations from Sections 4.1 and 4.2 was done to observe the effects of dual resonant frequencies. Similarly to Sections 4.1 and 4.2, the evaluation method used was based on the sample mean of the N*f*_ra_ and both samples (N*f*_rr_ and N*f*_ru_). The difference between the mean sample of N*f*_ra_ and both samples (N*f*_rr_ and N*f*_ru_) from the first peak and second peak were calculated. The ratio between the difference between sample to air (δ_unripe_ and δ_ripe_) was then analyzed and illustrated on the graph. Figure 14 shows the ratio between δ_unripe_ and δ_ripe_ for each coil turn configuration. From the graph, the 200-140 coil turn configuration provides the highest value as compared to the other two configurations. Detailed results of the ratio between δ_unripe_ and δ_ripe_ for each coil turn configuration is tabulated in Table 9.

### 4.4. Comparative Analysis to Previous Work

This paper presents a further analysis on the flat-type air coil structure that was presented in a previous paper [35], while specifically highlighting the effects of the dual resonant frequencies on the sensitivity of the induction-based oil palm fruit sensor. In [35] the effects of the air coil length and coil diameter of the air coil were investigated and evaluated the results in terms of the sensitivity of the sensor. The results in [35] suggested that the 5 mm air coil length with 0.12 mm coil diameter had the highest sensitivity value amongst all the other tested air coil structures. The 5 mm air coil length with 0.12 mm coil diameter was chosen in this comparative analysis as it has similar specifications as the air coil used in previous paper, with the exception to its coil turn configurations and numbers. The 5 mm air coil length with 0.12 mm coil diameter designated as single air coil in this paper revealed that the differences between the mean of ripe to unripe samples is 0.0112 [35].

**Figure 15.**
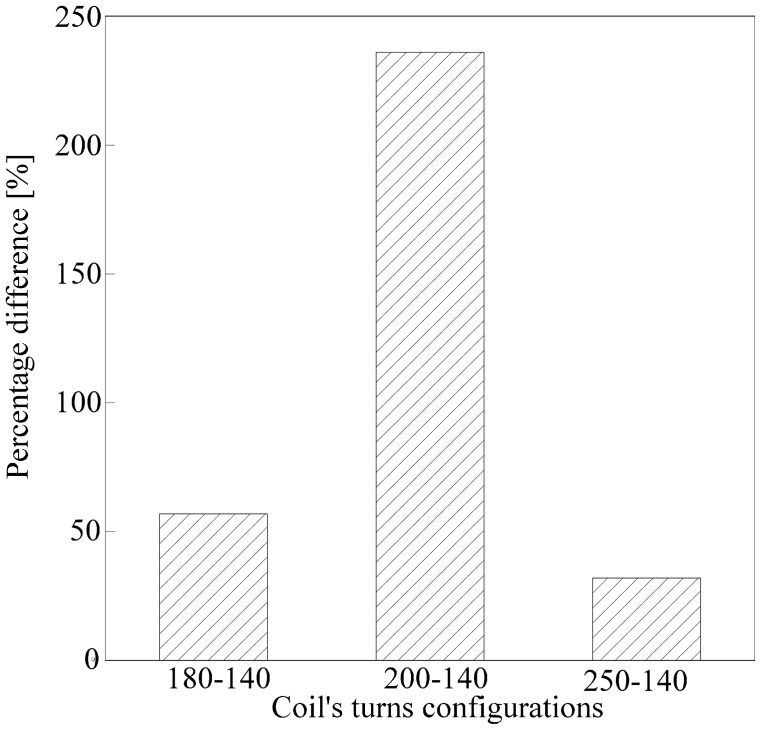
Percentage difference of the comparative analysis with the previous work [35].

In the beginning, the differences between the sample mean of the N*f*_rr_ and N*f*_ru_ and the ratio between δ_unripe_ and δ_ripe_ were calculated as in Sections 4.1 and 4.2. Then, the result was presented along with the results obtained from the dual peaks analysis from Section 4.2 in Table 10. There is a huge noticeable increase in the ratio between δ_unripe_ and δ_ripe_ as well as the differences between the sample mean of both samples in the 200-140 coil turn device used in this paper as compared to the previous work [35]. The results suggested an improvement of 371% in terms of the differences between the sample mean of both samples as compared to the previous work [35]. Detailed results in Table 10 are also presented as the percentage difference in terms of gap ratio shown in Figure 15. An increment of 236% is reported as an improvement from the previous works [35], as evident from the 200-140 coil turn configuration.

## 5. Conclusions

A dual flat-type shape air coil with numerous coil turn configuration dedicated to determining the maturity of oil palm fruit was designed and constructed. In this study, the investigation on the effects of the dual resonant frequencies appearing on the inductance characteristics of the air coil to the sensitivity of the oil palm fruit sensor was conducted. The sensitivity of the oil palm fruit sensor was examined in terms of two aspects. Firstly, the differences between the sample mean of both samples, ripe and unripe samples, as conducted in previous work [35] and secondly, the ratio between δ_unripe_ and δ_ripe_ was investigated. In this paper, the analysis on the dual resonant frequencies was done separately. As for the first peak analysis, the 200-140 coil turn configuration provided the highest ratio value of 2.648 and 0.0606 for the differences between the sample mean of both samples. Meanwhile, in the second peak analysis, the 250-140 coil turn device showed the highest ratio value of 1.847 with a slight difference among the other coil turn configurations. As for the differences between the sample mean between both samples, the value is 0.00990. Similarly, a ratio analysis between both peaks was also taken into consideration in this study. Results from the dual peaks analysis showed that the difference between the sample mean of both samples is 0.05281 and the ratio between δ_unripe_ and δ_ripe_ is 3.325 for the 200-140 coil turn configuration. Apart from that, a comparative analysis was done to analyze the improvement in sensitivity obtained in this study as compared to the previous study [35]. Results from this comparative analysis proved that the sensitivity of the inductive based oil palm fruit sensor using a dual flat-type shape air coil of 200-140 coil turn configurations was improved by 371% in terms of the differences between the sample mean of both samples and 236% in terms of ratio between δ_unripe_ and δ_ripe_. With further development and improvement in the air coil structure, the sensitivity of the inductive based oil palm fruit sensor could further improve and thus enhance the potential of the sensor to determine the maturity of the oil palm fruits in future work.

## Figures and Tables

**Table 1.** Coil turn configurations.

**Type**	**Number of Turns Used in the First Air Coil**	**Number of Turns Used in the Second Air Coil**
180-140	180	140
200-140	200	140
250-140	250	140

**Table 2.** Specification for frequency characteristics experimental setup.

**Parameter/Part**	**Value/Type**
Type of measurement setup	Series (*L*_s_–*R*_s_)
Voltage (V)	0.5
Frequency (MHz)	20–10
Sweep (points)	200
Coil diameter (mm)	0.12

**Table 3.** Characteristics of selected samples.

**Category**	**Surface Color**	**Age (WAA)**	**Number of Samples**
Unripe	Dark purple	After 7	15
Ripe	Red orange	18–21	15

**Table 4.** List of equations for the dual resonant characteristics.

**Item**	**Equation**	**Number**
The sample mean for *f*_r1a_	$\bar{f_{r1a}}=\overset{\sum_{1}^{n}fr1a}{\;}/\underset{n}{\;}$	(1)
The normalized resonant frequency of air sample of the first peak	$Nf_{r1a}=\overset{f_{r1a}}{\;}/\underset{\bar{f_{r1a}}}{\;}$	(2)
The normalized resonant frequency of ripe sample of the first peak	$Nf_{r1r}=\overset{f_{r1r}}{\;}/\underset{\bar{f_{r1a}}}{\;}$	(3)
The normalized resonant frequency of unripe sample of the first peak	$Nf_{r1u}=\overset{f_{r1u}}{\;}/\underset{\bar{f_{r1a}}}{\;}$	(4)
The sample mean for *f*_r21a_	$\bar{f_{r2a}}=\overset{\sum_{1}^{n}f_{r2a}}{\;}/\underset{n}{\;}$	(5)
The normalized resonant frequency of air sample of the second peak	$Nf_{r2a}=\overset{f_{r2a}}{\;}/\underset{\bar{f_{r2a}}}{\;}$	(6)
The normalized resonant frequency of ripe sample of the second peak	$Nf_{r2r}=\overset{f_{r2r}}{\;}/\underset{\bar{f_{r2a}}}{\;}$	(7)
The normalized resonant frequency of unripe sample of the second peak	$Nf_{r2u}=\overset{f_{r2u}}{\;}/\underset{\bar{f_{r2a}}}{\;}$	(8)

**Table 5.** Standard deviation and variance for ripe and unripe *vs*. air.

**Coil's Turns Configuration**	**σ_unripe_**	**σ_ripe_**	**σ^2^_unripe_**	**σ^2^_ripe_**
180-140	0.0100766	0.0493670	0.0021460	0.000181
200-140	0.0760790	0.0231407	0.0010063	0.000378
250-140	0.0120647	0.0274687	0.0008580	0.000413

**Table 6.** Differences between $\bar{Nf_{r1r}}$and $\bar{Nf_{r1u}}$ and the ratio between δ_unripe_ and δ_ripe_ for the first peak.

**Coil's Turns Configuration**	$\bar{Nf_{r1r}}-\bar{Nf_{r1u}}$	$\frac{\delta_{unripe}}{\delta_{ripe}}$
180-140	0.01639	1.726
200-140	0.06061	2.648
250-140	0.01785	1.491

**Table 7.** Standard deviation and variance for ripe and unripe *vs*. air.

**Coil's Turns Configuration**	**σ_unripe_**	**σ_ripe_**	**σ^2^_unripe_**	**σ^2^_ripe_**
180-140	0.025944	0.038155	0.0000216	0.0000132
200-140	0.010249	0.032475	0.0000270	0.0000011
250-140	0.031318	0.032144	0.0000297	0.0000214

**Table 8.** Differences between $\bar{Nf_{r2r}}$ and $\bar{Nf_{r2u}}$ and ratio between δ_unripe_ and δ_ripe_ for the second peak.

**Coil's Turns Configuration**	$\bar{Nf_{r2r}}-\bar{Nf_{r2u}}$	$\frac{\delta_{unripe}}{\delta_{ripe}}$
180-140	0.00530	1.691
200-140	0.00742	1.756
250-140	0.00990	1.847

**Table 9.** Differences between $\bar{Nf_{r1\_2r}}$ and $\bar{Nf_{r1\_2u}}$ and ratio between δ_unripe_ and δ_ripe_ for first peak and second peak.

**Coil Turn Configuration**	$\bar{Nf_{r1\_2r}}-\bar{Nf_{r1\_2u}}$	$\frac{\delta_{unripe}}{\delta_{ripe}}$
180-140	0.00675	1.552
200-140	0.05281	3.325
250-140	0.00693	1.304

**Table 10.** Comparative analysis of ratio between δ_unripe_ and δ_ripe_.

Coil's Turns Configuration	**Differences between** $\bar{Nf_{rr}}$ and $\bar{Nf_{ru}}$	$\frac{\delta_{\text{unripe}}}{\delta_{\text{ripe}}}$
Single air coil	0.01120	0.989
180-140	0.00675	1.552
**200**-**140**	**0.05281**	**3.325**
250-140	0.00693	1.304
